# Numerical approximation to the effects of the atmospheric stability conditions on the dispersion of pollutants over flat areas

**DOI:** 10.1038/s41598-021-89200-9

**Published:** 2021-06-02

**Authors:** J. I. Huertas, D. S. Martinez, D. F. Prato

**Affiliations:** 1 Energy and Climate Change Research Group, School of Engineering and Science, Tecnológico de Monterrey, Monterrey, Mexico; 2 Latin-American Center for Innovation and Logistics (CLI), Bogotá, Colombia

**Keywords:** Environmental impact, Atmospheric dynamics, Environmental sciences

## Abstract

Using the Computational Fluid Dynamics technique (CFD), we explored the effects of the atmospheric stability conditions on the dispersion of solid and gas-phase pollutants emitted from an area source located on a flat region. As an application, the dispersion of pollutants emitted from roads located on flat terrains was considered. Toward that end, we set up a model that describes the dispersion of air pollutants in a small region (< 1 km long) near the ground surface (< 250 m high). It consists of a neutrally stratified model modified to account for the atmospheric stability effects by imposing the near-ground stratification through the Monin–Obukhov similarity theory and the *k–ε* turbulence model adjusted for each atmospheric stability condition. Using this model, we simulated the dispersion of pollutants emitted from the road and plotted the resulting downwind concentrations in terms of dimensionless numbers. Results from our CFD-based model were highly correlated (*R*^2^ > 0.95) with the SF_6_ concentrations measured downwind a line source of this trace gas by the U.S. National Oceanic Atmospheric Administration in 2008 under different conditions of atmospheric stability. Numerical and experimental results showed that, under any of the stability conditions explored, the near-road pollutant concentrations are highly correlated (*R*^2^ > 0.87) to the concentrations observed under neutral conditions. When the atmosphere is extremely stable, those concentrations were up to 12 times higher than those observed under neutral conditions. We report the constant of proportionality obtained for every stability condition.

## Introduction

Environmental authorities and the scientific community are looking for micro-scale range models (~ 1 km) based on computational fluid dynamics (CFD) to evaluate the influence of the atmospheric stability conditions near the ground surface (~ 250 m) on the dispersion of pollutants from area sources like:

- Paved and unpaved roads,
- Uncovered areas exposed to the wind action, such as desertic areas, and particulate materials stored at open atmosphere (carbon piles as an example),
- Gaseous leaks from pipes (natural gas pipes as an example),
- Gases evaporated from pools exposed to open atmosphere (hydrocarbon spillages and sewer water as examples),
- Agro-industrial open burning.

These authorities want to use said models to (i) assess the exposure to air pollutants of the people living in the near distances to these sources of pollutants, (ii) to design countermeasures to mitigate their exposure, and (iii) to quantify the emissions from these diffusive sources of pollutants. However, the current most advanced pollutant dispersion models assume that the atmosphere is under neutral conditions^1^, despite the fact that experimental results show that atmospheric stability has a strong influence on the dispersion of pollutants^2,3^. This influence has not been quantified yet.

The physics phenomena occurring in the near-surface atmospheric boundary layer (SBL) under different atmospheric stability conditions are well known^4,5^. The condition of atmospheric stability is related to the change of temperature with height (the lapse rate) and wind speed^5^. When the vertical temperature gradient (*∂T/∂z*) is equal to the dry adiabatic lapse rate (*g/C*_*p*_ ~ − 9.8 K/100 km), the atmosphere is said to be under neutral conditions^6^. Neutral conditions only result when there is no heat transfer between the air and the ground, and therefore buoyancy effects are absent^4^. Heat transfer is the critical aspect of the non-neutral conditions. When the incoming solar radiation heats the earth's surface up, it warms the air in the SBL, creating buoyant forces that produce convective turbulence. As a result, the vertical profiles of temperature and wind speed are modified. In an unstable atmosphere, convection predominates, winds are usually weak, and therefore there is a strong vertical motion. A smoke plume leaving a source at the ground surface spreads rapidly, vertically, and horizontally. As mechanical turbulence increases, the atmosphere approaches the neutral condition, and the dispersion of the smoke plume decreases. Finally, the atmosphere becomes stably stratified when vertical mixing ceases, and mechanical turbulence is dampened. Under this condition, very little vertical dispersion of a smoke plume occurs. All these phenomena are of regional-scale nature (~ 100 km).

The condition of stability describes the degree of thermal turbulence in the atmosphere, and therefore its capacity of transporting and dispersing pollutants^5^. It can be defined as the tendency of the atmosphere to resist or enhance vertical motion, or alternatively, to suppress or augment the existing turbulence. Initially, the bulk Richardson number (*R*_*i*_)^6^ and the Monin–Obukhov Length (*L*)^7^ were used to define scales of the atmospheric stability conditions. However, these scales are hard to follow because they require the use of parameters that are difficult to measure, such as the surface heat transfer and the surface friction speed. Then, Pasquill and Gifford^8^ and later Pasquill and Turner^9^ defined categories of atmospheric stabilities based on the wind speed, solar radiation, and cloud coverage, which are parameters measured in meteorological stations. They classified the atmospheric stability into six categories: A for highly unstable or convective, B for moderately unstable, C for slightly unstable, D for neutral, E for moderately stable, and F for extremely stable. The main drawback of this scale is that it is a discrete scale. Table 1 compares these rating scales of atmospheric stability conditions.

**Table 1:** Parameters used to describe atmospheric stability conditions.

Atmospheric Stability Class	*Ri*^6^	*L*^7^	Pasquill–Gifford^8^
	–	(m)	
Neutral	*Ri* = 0	− ∞ < *L* < − 100	D
Slightly unstable	− 0.3 < *Ri* < 0	− 100 < *L* < − 10	B and C
Extremely unstable		− 10 < *L* < 0	A
Extremely stable	*Ri* > 0.25	0 < *L* < 20	F
Slightly Stable	0 < *Ri* < 0.25	20 < L < ∞	E

Wind speed	Daytime			Nighttime	
	Solar radiation (W m^−2^)			Cloud cover	
(m/s)	> 580	290–580	< 290	> 50%	< 50%
< 2	A	A–B	B	E	F
2–3	A–B	B	C	E	F
3–5	B	B–C	C	D	E
5–6	C	C–D	D	D	D
> 6	C	D	D	D	D

Richardson number $Ri=\frac{g}{\rho }\frac{\partial \rho /\partial z}{{\left(\partial u/\partial z\right)}^{2}}$ Monin–Obukhov Length $L=\frac{{u}_{*}^{3}}{k\left(g/{T}_{0}\right)\left({H}_{0}/\rho {c}_{p}\right)}.$ Where , *ρ*, and *C*_*p*_ are the air thermal conductivity, density, and specific heat, respectively. *T*_*o*_ and *H*_*o*_ are the surface temperature and heat flux, respectively. *z* is height, and *g* is the gravity constant. *u* and u* are the wind and friction velocity, respectively. According to the Pasquill–uifford stability classification classes *D* applies to heavily overcast skies, at any wind speed day or night.

Few studies have been conducted to experimentally quantify the effects of the atmospheric stability condition on pollutant dispersion. Zoras et al.^10^ used a two-year period of meteorological and PM_10_ concentration data from a Greek region (50 km^2^) where a coal plant was the main source of pollutants. They observed that most of the PM_10_ episodes (42.6%) occurred when the atmosphere was extremely stable (F). In 2008, aiming to quantify the effects of roadside barriers on the downwind dispersion of pollutants emitted by road sources, the National Oceanic Atmospheric Administration (NOAA) carried out the NTRS08 campaign, where they dispersed SF_6_ and measured the concentration of this trace gas at several positions downwind a solid barrier. They observed that the SF_6_ concentrations downwind, with and without the barrier, were higher when the atmosphere was stable compared to when it was neutral or unstable^11^. However, they did not quantify that magnification effect. In the same year (2008), the Environmental Monitoring Center of Lanzhou (China) monitored for 11 months the meteorological variables and pollutant concentrations at three contrasting sites while using the radon-based technique to monitor atmospheric stability. They assumed that the emissions in the city remained constant throughout the year and compared the early morning air pollutant concentrations to eliminate the effects of the diurnal changes in the mean mixing depth. They found that the morning peaks of pollutants concentrations increased by a factor of 2–5 from the highly unstable to the stable atmospheric conditions^12^. All these experimental works agree that the pollutants concentrations are higher under stable atmospheric conditions than under unstable conditions. However, the magnitude of this effect varies among experimenters. Furthermore, these variations could be due to the sole effect of variations in wind speed rather than variations in the atmospheric stability conditions.

The effects of the atmospheric stability conditions on pollutant dispersion can also be studied analytically. The physics phenomena occurring under the different atmospheric stability conditions are described by the mass, momentum, and energy conservation equations^5^. Nevertheless, the task of solving those equations is challenging because they are nonlinear partial differential equations.

Authors have faced this challenge by solving these governing equations with a high time and space resolution to capture the turbulence phenomena (high-frequency fluctuations in speed) at the mesoscale range. In this way, they grasp the short- and long-range turbulent eddies of the different atmospheric stability conditions. This approach is known as direct numerical simulation (DNS). The CFD engineering and geophysical modeling communities have converged in that this is the best approach to model the atmosphere dynamics^13^. However, this approach is computationally expensive and has not been used to study the dispersion of pollutants systematically.

There are several challenges when attempting to use this DNS alternative for the study of the dispersion of pollutants. The main ones are related to the differences in the spatial and temporal scale of the atmospheric dynamics and of the dispersion phenomena:

- The area of interest in the study of pollutant dispersion is near the emission source, where concentrations are the highest. i.e., the interest is at the micro-scale level (< 1 km). Then, the challenge is to reproduce the atmosphere dynamics, which is a regional phenomenon (~ 100 km), in a reduced computational domain.
- The time scale of interest in the study of pollutant dispersion is of hours or days. Time average concentrations are of interest rather than instantaneous concentrations. Then, the challenge is to obtain long-term concentrations at a reasonable computational time.

Some authors have proposed the coupling between meso- and micro-scale models where results from the mesoscale model are used as boundary conditions to the micro-scale model^14^. However, the issues described above remain still unresolved, at least for practical applications^15^.

As an alternative to the DNS approach, Reynolds (1895), working at the micro-scale range, described the instant velocity as a short-time-average velocity plus a fluctuating velocity. Then, he transformed the instant governing equations in terms of these two velocity components, which produced additional unknown variables termed as Reynolds stresses. Those stresses represent turbulent transport of momentum and energy. Usually, it is assumed that those stresses can be expressed as a linear combination of the velocity gradients. However, additional equations are still needed to close the description of the turbulence problem. Turbulence models based on this approach are known as Reynolds Average Navier Stokes (RANS) models. Among them, the *k–ε* turbulence model has been the most widely used. The *k–ε* model adds an equation for the turbulent kinetic energy (*k)* and one equation for the turbulent energy dissipation rate (*ε)*. Adding these turbulence models to the momentum and energy equations, CFD engineers have been able to reproduce the experimental observations related to the interaction of the flow field with solid bodies in terms of pressure gradients (aerodynamics) and in terms of heat and mass transfer.

All these turbulence models do not reproduce the physics of the turbulence phenomena, but they simulate the average turbulence effects on the flow field. The main drawback of this approach is that there is not a single model for every application. Furthermore, every turbulence model must be adjusted to the specific application via experimental calibration.

Following this approach, the *k–ε* turbulence model has been used to reproduce the effects of the atmospheric dynamics on the average flow field on the SBL^16^. Most of the work developed simulating near-road air pollution at the microscale level (< 1 km) adopts this alternative. However, these studies assume uniform temperature and isotropic turbulence, which are features of a neutral atmosphere^17–19^. Using this alternative, Huertas and Prato^17^ used meteorological data as input to their Near-Road CFD (NR-CFD) model and simulated the hourly dispersion of particles near two unpaved roads located on a flat region. They obtained daily and monthly average values of particle concentration that were highly correlated to measurements obtained at several points downwind the roads.

The CFD based models that adopt the neutral atmosphere assumption have been criticized for not including the atmospheric dynamics and its different conditions of stability. It has also been counter-argued that in the very near ground surface, the flow field is highly influenced by the mechanical turbulence generated by the presence of physical obstacles (e.g., buildings), which disturb the free wind flow, while the atmospheric stability conditions are mesoscale or regional phenomena^20^, and therefore its influence on the dispersion of pollutants is minor at the very near ground surface.

CFD-based models can be used to evaluate the effects of the different atmospheric stability conditions on the dispersion of pollutants systematically. However, few works have been conducted with this objective in mind. Using CFD, Pieterse et al.^21^ evaluated the effects of three atmospheric stability conditions on the wind flow over flat terrain. They found the parameters for the *k–ε *turbulence model that best describe those atmospheric conditions. However, they did not use their model to study the effects of atmospheric stability on the dispersion of pollutants. Steffens et al.^18^ replicated in CFD the experimental results of the NRTS08 campaign described above. They modeled the atmospheric stability conditions by implementing the Monin Obukhov Similarity (MOS) theory and evaluated the performance of two turbulence models: Reynolds Average Navier Stokes (RANS) and Large Eddie Simulation (LES). They found that both models produced similar results. Nevertheless, they did not quantify the effect of the atmospheric stability conditions on the dispersion of the SF_6_ tracer gas. The coupling of the MOS theory with the *k–ε* turbulence model produces a rough description of the atmospheric dynamics in the SBL, which could be useful for applications likes the study of the dispersion of pollutants and for the wind engineering community. However, we highlight that this approximation is not intended for the study of the atmospheric dynamics.

We propose to advance Pieterse's work, and use said description of the atmosphere dynamics to study the effects of the different stability conditions on the dispersion of pollutants. Specifically, the objective of this work is to quantify the effects of atmospheric stability conditions on the dispersion of pollutants downwind an emission source, such as roads, located in flat regions.

In the process of pursuing this objective, the following contributions to new knowledge were developed:

- The proposed approach was implemented in the NR-CFD model. Results from said model reproduces the experimental measurements obtained in the NRTS08 campaign studying the dispersion of a line source of SF_6_ under different atmospheric stability conditions (Fig. 4b). In this manuscript, the NR-CFD model is described in a way that it could be reproduced by others.
- An approximation to the quantification of the effects of the atmospheric stability conditions on the ground pollutant concentration downwind an area emission source was obtained by systematically using the NR-CFD model and experimental data (Fig. 5f).
- This manuscript reports that the ground pollutant concentration downwind an emission source under any atmospheric stability condition is highly correlated to the concentration observed under neutral conditions (Fig. 5e–g) when it is expressed in terms of dimensionless numbers (C* vs. x*). Furthermore, that said C* vs. x* profile is independent of variations in the wind speed, mass emission rate, and of the pollutant's nature.

## Methodology

Aiming to study the effects of the atmospheric stability conditions on the dispersion of the pollutants emitted from an area source located over a flat surface, (i) we selected the simplest possible representative case. i.e., a road on a horizontal area without any obstacle to the wind flow. Then, (ii) an approximation to the physics occurring in the atmosphere very near the ground surface (< 250 m high), in a small domain (< 1 km long), was implemented, by solving via CFD the mass, momentum, and energy equation, coupled to the MOS theory and the appropriate *k–ε* turbulence model for each atmospheric stability condition. (iii) Using this model (NR-CFD model), the dispersion of pollutants emitted from a road was simulated and the resulting downwind concentrations were expressed in terms of dimensionless numbers. (iv) Results from the NR-CFD model were compared with the experimental measurements obtained in the NRTS08 campaign. (v) Finally, the experimental and simulated results were used to compare the concentrations downwind the road under different atmospheric stability conditions with the ones observed under neutral conditions. Next, we will describe each of these steps.

### Case of study

**Figure 1:**
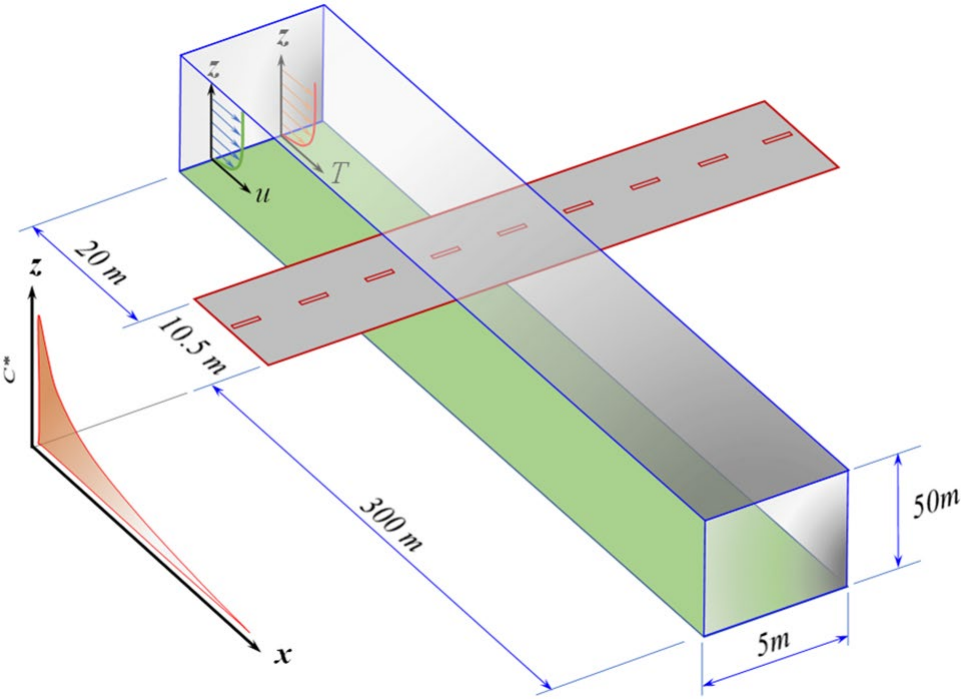
Computational domain used in this study. The length scale is not uniform on the illustration.

In this work, we studied the dispersion of the pollutants emitted from a road located on a flat region without the presence of any obstacle to the wind free flow (Fig. 1). As stated before, this case is an illustrative example of a more general case, which is the dispersion of the pollutants emitted from an area source located over a flat surface.

We are interested in the region near the source where the highest concentrations occur. This means that the interest is located very near the ground surface (*z* < 250 m) and close to the emission source (length < 1 km). For this particular case, we defined the computational domain shown in Fig. 1, which is a box of dimensions 5 m, 330.5 m, and 50 m in the crosswind, along-wind, and vertical direction, respectively. These dimensions were selected according to Franke et al.^22^, who recommended the minimum dimensions of the computational domain in the way that the walls do not interfere with the dispersion of the pollutants.

### Simulation of the atmosphere dynamics

Next, we describe our approach to simulate, at microscale level (length < 1 km), the physics occurring in the near-surface atmospheric boundary layer (height < 250 m), under different stability conditions, using state of the art CFD solvers.

The first step in the CFD simulation of pollutant dispersion near roads is the simulation of the atmospheric dynamics. The physics phenomena occurring in the atmosphere are described by the well-known mass, momentum, and energy governing equations^5^. These equations can be solved numerically using the computational fluid dynamics (CFD) technique. In this technique, the space domain is divided into small volumes, and these equations are solved over each volume. Besides the discretization of the computational domain into a large number of finite volumes, the simulation of the physics phenomena occurring in the atmosphere requires the specification of the conditions under which those equations need to be solved.

In the study of dispersion of pollutants, as in several other applications, researchers are interested in short (1 h) and long-term (~ 1 year) average results. Implicitly, this interest involves a pseudo-steady-state approximation where the daily evolution of the atmosphere is studied as a set of successive states where at each time interval, the atmosphere is assumed to be under a steady-state condition. This assumption is acceptable if the time step of the simulation is smaller than the response time of the SBL to changes in radiative forcing. The SBL's response to changes in surface radiative forcing is a large-scale phenomenon with long response times (~ 1 h)^6,23,24^. In practice, 1-h intervals are suitable, as meteorological data is reported in this way.

When modeling the atmospheric dynamics over a flat surface without any perturbation to the wind free flow, this pseudo steady state assumption implies:

- The condition of horizontal homogeneity. The steady-state assumption of the atmosphere over a flat surface within a small computational domain implies that the value of any property remains the same at any position with the same height. It requires that, in the absence of any perturbation, the vertical wind and temperature profiles at the inlet remain the same at the outlet. This condition of horizontal homogeneity agrees with the observation that at any given time, for example, the ground temperature is the same everywhere within the microscale domain.
- The inlet boundary condition is known and they satisfy the governing equations. The steady-state assumption requires that at every vertical position at the inlet, the value of each variable is a known input data. The use of constant values or of any arbitrary function for the physical properties at the inlet will lead to a violation of the horizontal homogeneity condition. Some researchers^18^ have measured the vertical wind and temperature profiles and used them as the inlet boundary condition. However, in practice, researchers only have available meteorological data, where wind speed and temperature are measured at a single height at each time interval. Later in this section, we will describe how the inlet input data can be obtained from the meteorological data.
- Agreement between the inlet and the ground boundary conditions. The condition of horizontal homogeneity requires that the ground boundary conditions for momentum and heat transfer agree with the inlet boundary condition. Otherwise, wind speed and air temperature will change along with the computational domain.

#### Inlet boundary condition

The objective is to specify the inlet boundary condition based on the measurements of temperature and wind speed at 10 m above ground, in a way that the result of the simulation of the atmospheric dynamics, under the steady-state assumption, satisfies the homogeneity condition. That is, the vertical inlet profile for wind speed and temperature at the inlet and outlet should be the same.

The first alternative is to start with any arbitrary vertical profile for wind speed and temperature and simulate the wind flow over an extremely long flat surface subject to a desired constant ground heat transfer condition. The resulting profiles will satisfy the conditions specified above. This process is cumbersome, especially since it should be carried out for each time-step of meteorological data.

The second alternative is to use the knowledge gained on the atmospheric dynamics to specify the inlet boundary condition. In the 1920s, studying the fluid flow on a flat surface, Von Karman observed that the fluid flow on that surface exhibited a parabolic profile and that when the said profile is expressed in terms of dimensionless numbers, it does not depend on the downwind position. Monin–Obukhov, in the 1950s, extended Von Karman's concepts of fluid mechanics to the modeling of the atmosphere dynamics. They specified the shape of wind speed profiles for several stability conditions (Eqs. 1–5). Today that approach is known as the Monin–Obukhov similarity (MOS) theory. It states that any mean flow quantity in the SBL (e.g., momentum or energy), when normalized by an appropriate scaling parameter, is a unique function of $\zeta =z/L$ where $\zeta$ is termed as the buoyancy parameter^5^, and *L* is the Monin–Obukhov length, which describes the atmospheric stability condition (Table 1).

Based on the observation that (i) in the SBL the wind speed (*u*) varies logarithmically with height and that (ii) the surface roughness forces the mean wind speed to be zero at the ground surface, the MOS theory states that the vertical profile for wind speed in the SBL follows Eq. (1)^23^.

1 $$u_{\left( z \right)} = \left( {\frac{{u_{*} }}{\kappa }} \right)\left[ {ln\left( {\frac{z}{{z_{0} }}} \right) - \psi_{m} \left( \frac{z}{L} \right)} \right]$$

where u* is the friction velocity, which is calculated from ${u}_{*}=\sqrt{{\tau }_{w}/\rho }$; *k* is the Von Karman constant which has a value of 0.41^5^; and *z*_*o*_ is the surface roughness that for the case of short grass, the recommended value is 0.03 m^5^. ${\psi }_{m}$ is the similarity function for the flux of momentum (Eqs. 3 and 4). For 0 < *z* < *z*_*o ,*_ the wind speed is zero. In Eqs. (4) and (5), $\alpha ={(1-15z/L)}^{1/4}$. We selected a set of values for *L* and used the resulting vertical profiles of wind speed as the inlet boundary condition.

The MOS theory also specifies the vertical profiles for the ambient temperature (Eq. 2,^25^). In this case *θ* is the potential temperature which is calculated from $\theta =T{\left(P/{P}_{0}\right)}^{R/{c}_{p}}$; *θ*_*o*_ is the ground potential temperature; θ* is the potential temperature which most of the time is equal to *T*_*0*_, and ${\psi }_{h}$ is the similarity function for heat flux described by Eqs. (3) and (5). We used these profiles as the inlet boundary condition of the computation domain.

2 $$\theta \left( z \right) - \theta_{0} = \left( {\frac{{\theta^{*} }}{\kappa }} \right)\left[ {ln\left( {\frac{z}{{z_{0} }}} \right) - \psi_{h} \left( \frac{z}{L} \right)} \right]$$

3 $$\psi_{m} = \psi_{h} = - 5\frac{z}{L}\quad {\text{for}}\;\varsigma \ge {0}$$

4 $$\psi _{m} = \ln \left[ {\left( {\frac{{1 + \alpha ^{2} }}{2}} \right)\left( {\frac{{1 + \alpha }}{2}} \right)^{2} } \right] - 2\tan ^{{ - 1}} \alpha + \frac{\pi }{2}\quad {\text{for}}\;\varsigma < {0}$$

5 $$\psi _{h} = 2\ln \left( {\frac{{1 + \alpha ^{2} }}{2}} \right) \quad {\text{for}}\;\varsigma { < 0}$$

Under stable atmospheric conditions, both profiles tend to become linear for large values of $\zeta$. Under unstable conditions ${\psi }_{m}$ and ${\psi }_{h}$ are positive, and therefore, the velocity and temperature profiles in the surface layer are more curvilinear as the condition of instability increases^5^.

#### Use of the wall function for the ground boundary condition

The flow field over the ground surface should satisfy the non-slip condition. The first alternative to implement this condition is to specify a wind speed equal to zero at the surface and let the model to solve for the vertical wind speed. This alternative requires finite volumes much smaller than the boundary layer thickness, which is expensive computationally speaking.

Instead, best practices in CFD recommends the use of the near-wall treatment. This second alternative uses again the knowledge gained on fluid dynamics to specify the vertical wind speed profile as the ground boundary condition. It reduces substantially the number of elements required near the surface. Best practices in CFD suggests that the size of the elements in the ground surface should be at most half of the estimated boundary layer. CFD modelers developed the metric *y*^+^ to define the size of the elements near the surface to assure congruency of the boundary condition with the solution above the ground surface. Following this practice, the simulation of wind flow over a grass surface required finite volumes of ~ 30 cm near the ground surface, which is equivalent to a *y*^+^ value of 214.2. This value satisfies the log-law of modeling that recommends 30 < *y* +  < 1000^26^. To fulfill the horizontal homogeneity condition, we used as ground boundary conditions the same velocity profile used at the inlet boundary condition. Similarly, we used the vertical profile of temperature as the ground boundary condition for the energy equation.

#### Other boundary conditions

As described before, the height of the computational domain should be selected as the minimum where vertical interaction (other than turbulences) is negligible. Then, following the best CFD practices, we selected as the upper boundary condition the symmetry condition (zero gradients). When simulations were carried out in 3D, we proceeded in a similar way and used the periodic boundary condition, which is used when the solution in the crosswind direction remains the same with periods equal to the width of the computational domain. There was no need to specify any surface roughness at the road because it was modeled as a source of pollutants. That is, we specified a uniform velocity inlet at the road. Physically it means that the incoming wind flow faces a perpendicular ascending wind flow with a high pollutant concentration.

#### Turbulence model

As stated in the introduction section, the governing equations for momentum and energy need to be solved with a high time and space resolution to capture the turbulence phenomena (high-frequency fluctuations in speed). This approach is known as direct numerical simulation (DNS), which is computationally expensive. As an alternative, a model that describes the turbulence phenomena is added to the momentum and energy equations. Turbulence models for the SBL should account for both shear and buoyancy produced turbulence^21^. The *k–ε* turbulence model meets this requirement and has been widely used in SBL-related studies^16^. The *k–ε* model adds an equation for the turbulent kinetic energy (*k,* Eq. 6) and an equation for the turbulent energy dissipation (*ε,* Eq. 7). Both ensure the closure of the system of equations that describe the physics phenomena occurring in the SBL.

6 $$\frac{\partial }{\partial t}\left( {\rho k} \right) + \frac{\partial }{{\partial x_{i} }}\left( {\rho ku_{i} } \right) = \frac{\partial }{{\partial x_{j} }}\left[ {\left( {\mu + \frac{{\mu_{t} }}{{\sigma_{k} }}} \right)\frac{\partial k}{{\partial x_{j} }}} \right] + G_{k} + G_{b} - \rho \varepsilon - Y_{M} + S_{k}$$

7 $$\frac{\partial }{\partial t}\left( {\rho \varepsilon } \right) + \frac{\partial }{{\partial x_{i} }}\left( {\rho \varepsilon u_{i} } \right) = \frac{\partial }{{\partial x_{j} }}\left[ {\left( {\mu + \frac{{\mu_{t} }}{{\sigma_{\varepsilon } }}} \right)\frac{\partial \varepsilon }{{\partial x_{j} }}} \right] + C_{1\varepsilon } \frac{\varepsilon }{k}\left( {G_{k} + C_{3\varepsilon } G_{b} } \right) - C_{2\varepsilon } \rho \frac{{\varepsilon^{2} }}{k} + S_{\varepsilon }$$

In these equations, *G*_*k*_ (Eq. 8) represents the generation of turbulent kinetic energy due to the mean velocity gradient, and *G*_*b*_ (Eq. 9) is the generation of turbulent kinetic energy due to buoyancy. *Y*_*M*_ represents the contribution of the fluctuating dilatation in compressible turbulence to the overall dissipation rate. *µ*_*t*_ is the turbulent viscosity, and ${C}_{1\varepsilon }$, ${C}_{2\varepsilon }$ and ${C}_{3\varepsilon }$, are constants. ${\sigma }_{k}$ and ${\sigma }_{\varepsilon }$ are the turbulent Prandtl numbers for *k* and $\varepsilon$, respectively. *S*_*k*_ and *S*_*ε*_ are source terms of *k* and *ε*^27^.

8 $$G_{k} = \tau_{ij} \frac{{\partial u_{i} }}{{\partial x_{j} }} = - \rho \overline{{u_{i}^{^{\prime}} u_{j}^{^{\prime}} }} \frac{{\partial u_{i} }}{{\partial x_{j} }}$$

9 $$G_{b} = \beta g_{i} \frac{{\mu_{t} }}{{Pr_{t} }}\left( {\frac{\partial \theta }{{\partial x_{i} }}} \right) = \beta g_{i} \frac{{\mu_{t} }}{{Pr_{t} }}\left( {\frac{\partial T}{{\partial x_{i} }} - \frac{{g_{i} }}{{c_{p} }}} \right)$$

*τ*_*ij*_ is the shear stress in the direction *i* and perpendicular to the plane *j*; *u'*_*i*_ is the fluctuating wind speed in the *i* direction; *β* is the fluid thermal expansion coefficient; *T* is temperature, and *Pr*_*t*_ is the energy turbulent Prandtl number^27^. In Eq. (9), ${g}_{i}/{c}_{p}$ is the adiabatic lapse rate. An unstable condition will occur when the actual $\partial T/\partial {x}_{i}$ is greater than the ${g}_{i}/{c}_{p}$. When $\partial T/\partial {x}_{i}$ is less than ${g}_{i}/{c}_{p}$, turbulence will be suppressed from the atmosphere, and the SBL will exhibit a condition of stability^16^.

Pieterse and Harms^22^ measured *k* and *ε* at different heights for different atmospheric stability conditions on flat terrain without any obstacle to the wind flow. Then, they simulated the wind flow via CFD. They reproduced those experimental results fixing average values for *k* and *ε* at the inlet boundary conditions (Table 2) and specifying values for ${C}_{3\varepsilon }$ within the computational domain that depends only on the vertical direction. Alinot and Masson^16^ expressed the ${C}_{3\varepsilon }$ obtained as a polynomial expression (Eq. 10) that depends on the height (*z*). In Eq. (10), *L* is the Monin–Obukhov length, *n* is an integer, and *a*_*n*_ is the coefficient found by Alinot and Masson^16^. ${C}_{1\varepsilon }$ and ${C}_{2\varepsilon }$ remained constant. We adopted this methodology in our work to describe the variations of turbulence with atmospheric stability conditions, and we also considered the *k* and *ε* average values reported by them for the inlet boundary condition.

10 $${\text{C}}_{{{\upvarepsilon }3}} = \mathop \sum \limits_{{{\text{n}} = 0}}^{5} {\text{a}}_{{\text{n}}} \left( {\frac{{\text{z}}}{{\text{L}}}} \right)^{{\text{n}}}$$

**Table 2:** Parameters used to describe atmospheric stability conditions.

Atmospheric stability class	*T*_*o*_	u_*_	*k*	$\varepsilon$
	K	m s^−1^	m s^−2^	m^2^ s^−2^
Extremely unstable	313	0.497	5.5069	0.003368
Slightly unstable	305	0.485	5.5069	0.003368
Neutral	298	0.530	1.2699	0.000544
Slightly stable	283	0.472	1.1089	0.003838
Extremely stable	280	0.467	1.1089	0.003838

### Simulation of the dispersion of pollutants

The transit of vehicles over roads generates pollutants that are emitted from the vehicle's tailpipe and from the road-wheels interaction. Due to the complexity of the near-road air pollutions, researchers have divided its study into 3 phases^15^:

- From the source (tailpipe or wheel-road interaction) to the ambient near the vehicle, where solid and gas phase pollutants maintain their characteristics with a dilution ratio of up to 1000:1.
- From the vehicle to the road where pollutants mix with other sources of pollutants with a dilution ratio of up to 10:1.
- From the road to the ambient downwind the road.

Our work concentrates on the last phase. We, as all studies focused on the third phase, assume uniform concentration on the road as a net result of the multiple vehicles crossing by the same position over a long time, regardless of the exact source of the pollutants (tailpipe or road-wheel interaction). For comparative purposes, we chose an arbitrary emission of *E*_*b*_ = 1 g s^−1^ m^−2^ and verified that the results do not depend on the emission rate.

Chemical reactions were not considered in the model, which is an acceptable assumption for the case of inert pollutants or pollutants with a mean lifetime much longer than the residence time within the computational domain. For a wind speed of 1 m s^−1^, the residence time of the pollutants within the computational domain (Fig. 1) is ~ 5.5 min.

Based on the experimental work of Huertas et al.^28^, we modeled total suspended particles (TSP) that follow a Rossin-Ramler particle size distribution with a maximum diameter of 34 μm, a minimum diameter of 1 μm, an average diameter of 8.5 μm and a dispersion parameter of 3. We also used the Discrete Random Walk (DRW) model to observe the particle dispersion. This model is a stochastic zeroth-order tracking method that starts with the velocity flow field obtained by solving the Navier–Stokes equations. Then, the DRW integrates Eqs. (11) and (12) to predict particle trajectory using the local continuous phase conditions as the particle moves through the flow, for a sufficient number of representative particles^27^.

11 $$\frac{{d{\varvec{u}}_{{\varvec{p}}} }}{dt} = {\varvec{F}}_{D} + \frac{{{\varvec{g}}_{z} \left( {\rho_{p} - \rho } \right)}}{{\rho_{p} }} + {\varvec{F}}$$

12 $${\varvec{F}}_{D} = \frac{18\mu }{{\rho_{p} d^{2} }} \frac{{C_{d} Re}}{24}\left( {{\varvec{u}} - {\varvec{u}}_{p} } \right)$$

In Eqs. (11) and (12), ***u*** and ***u***_*p*_ are the fluid and particle velocity vectors, ***F***_*d*_ is the drag force acting on the particle, ***g***_*z*_ is the gravity vector acting in the vertical direction, *ρ* and *ρ*_*p*_ are the fluid and particle density, ***F*** is any external force acting on the particle, *d* is the aerodynamic particle diameter, *Re* is the Reynolds number based on the local speed and particle diameter, *µ* is the fluid viscosity, and *C*_*d*_ is the particle drag coefficient. Bold symbols are vectors.

The DRW model does not include particle diffusion effects since said phenomena is non-significant for the size of the particle considered in this study (0.1 < *d* < 30 µm)^27^. When particles become smaller than the ones considered in this work (ultra-fine particles, *d* < 100 nm), Eqs. (11) and (12) are modified to consider molecular effects. This DRW model determines the effect of the particles on the velocity flow field of the continuous phase via the source terms of momentum, and iterates, coupling the impact of the discrete and the continuous phases, on the fluid flow. We used the discrete phase module of ANSYS –Fluent v17, to simulate the dispersion of the particles described above. This module has implemented the DRW described above^27^. We used 50.000 time-steps to track the trajectory of each particle within the computational domain. Finally, we established that particles that reach the ground surface get trapped in the short grass that covers the near road surface.

#### Convergence and grid independence analysis

In this study, the pressure-based solver of ANSYS-Fluent v17.1 was used, with the following options: (i) Double precision, as recommended for large geometries and significant pressure and speed variations^29^. (ii) The steady-state transport equations. (iii) The second-order interpolation scheme. (iv) The semi-implicit method for pressure-linked equations (SIMPLE), which uses a combination of continuity and momentum equations to derive an equation for pressure (or pressure correction).

Quad-type elements were used to discretize the domain shown in Fig. 1. Aa structured mesh was applied because it is easy to implement, requires less computing time, and facilitates particle tracking. We used 3.4 million of computational cells for the simulations with refinement in the regions of interest, such as the sections adjacent to and downwind the roadway. This mesh was selected after a grid independence analysis from 0.11 to 3.9 million elements. On a server with 16 parallel processors, solution times ranged from 30 min to 3 h for the finest mesh.

### Use of dimensionless numbers to study the effect of stability conditions on pollutants dispersion

Wind speed has a strong influence on pollutants dispersion. Aiming to isolate the effects of wind speed from the effects of the other variables that influence the atm(ospheric stability condition, results are reported in terms of the dimensionless numbers developed by Huertas and Prato^17^. They demonstrated that, for neutral atmospheres, when pollutant concentrations downwind the road are expressed in terms of the dimensionless numbers for concentration (C*, Eq. 13) and distance to the road (x*, Eq. 14), the resulting profiles are independent of wind speed (*U*), emission rates per unit area (*E*) and the nature of the pollutant through the Schmidt number (Sc). Therefore, variations on the obtained universal profile are attributed to variations on the atmospheric stability conditions rather than just variations on wind speeds.

13 $$C^{*} = \frac{C U}{E}S_{c}$$

14 $$x^{*} = \frac{x}{w}$$

In Eqs. (13) and (14), $w$ is the road width, and *S*_*c*_ is the ratio of momentum diffusivity (kinematic viscosity, υ) and mass diffusivity (*D*). It is used to characterize fluid flows in which there are simultaneous momentum and mass diffusion-convection processes.

### Comparison with experimental results

**Figure 2:**
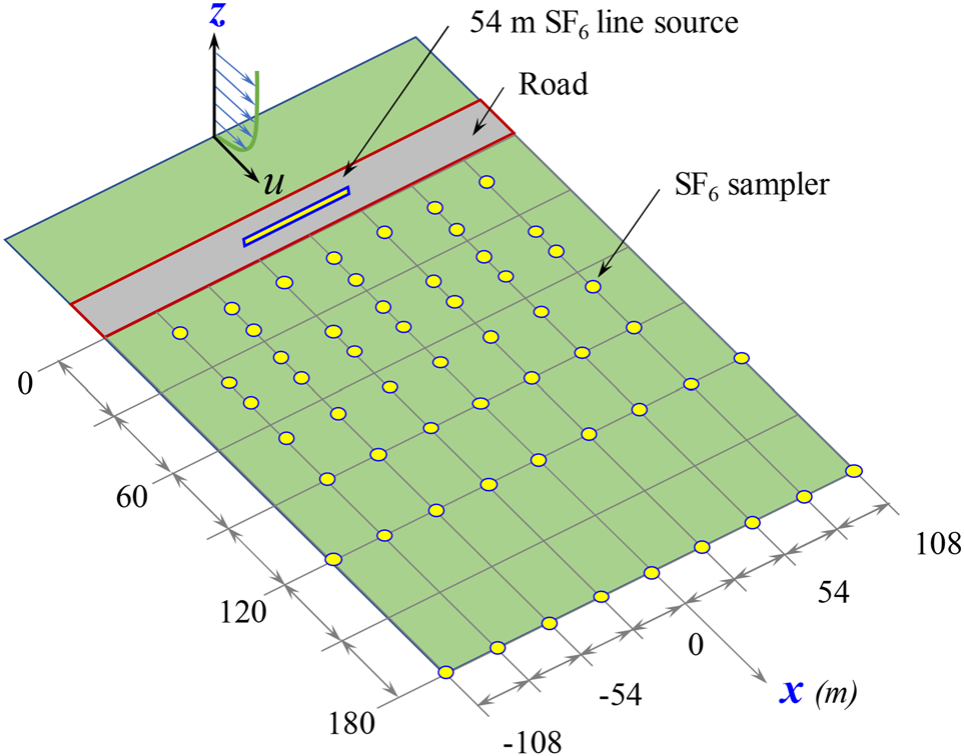
Illustration of the experimental setup implemented in the NTRS08 campaign in Idaho Falls, USA. In this study, we used the experimental results obtained without the barrier.

Aiming to validate the implemented CFD-based model for the dispersion of pollutants near roads (NR-CFD model), results obtained from this model were compared with the experimental data obtained in the Near Road Tracer Study (NRTS08). This study was carried out in 2008 by the NOAA, INL, ARL, and EPA in Idaho Falls, USA^30^ and reported by Finn et al.^11^ and Steffens et al.^18^. Figure 2 illustrates the experimental setup implemented. They released a 1 g s^−1^ constant flow of a tracer gas (SF_6_) distributed uniformly along a 54 m line and used an array of bag samplers to measure the 15 min average SF_6_ downwind concentrations at 3, 4, 6, 8, 11,15, 20, and 30 times the road width. They adopted an equivalent road width of 6 m in their experiments. This gas was chosen due to its negligible background concentration in the atmosphere. They placed anemometers at several vertical positions to measure wind speed, wind direction, turbulence characteristics, friction velocity, heat flux, and atmospheric stability. Tests were repeated under several atmospheric conditions when the wind flowed nearly perpendicular to the line emission source.

Even though their interest was the study of the effects of a side road barrier on the dispersion of pollutants, they carried out 31 tests without any barrier to the wind free flow under different atmospheric stability conditions. We used this subset of data obtained without any barrier for our comparative analysis. A Schmidt number for SF_6_ of 0.207 was used to express their experimental results in terms of C* (Eq. 13).

## Results and discussion

As a first step, we ran the NR-CFD model without any emission from the road for the stability conditions considered in this study, observed the evolution of the wind speed and vertical temperature profiles along with the computational domain, and confirmed that they remained essentially unaltered. This fact confirms that the condition of horizontal homogeneity for wind speed and temperature were met. We highlight that this condition of horizontal homogeneity is met when the inlet and ground boundary conditions are congruent with each other and when those boundary conditions satisfy the governing equations of momentum and energy.

**Figure 3:**
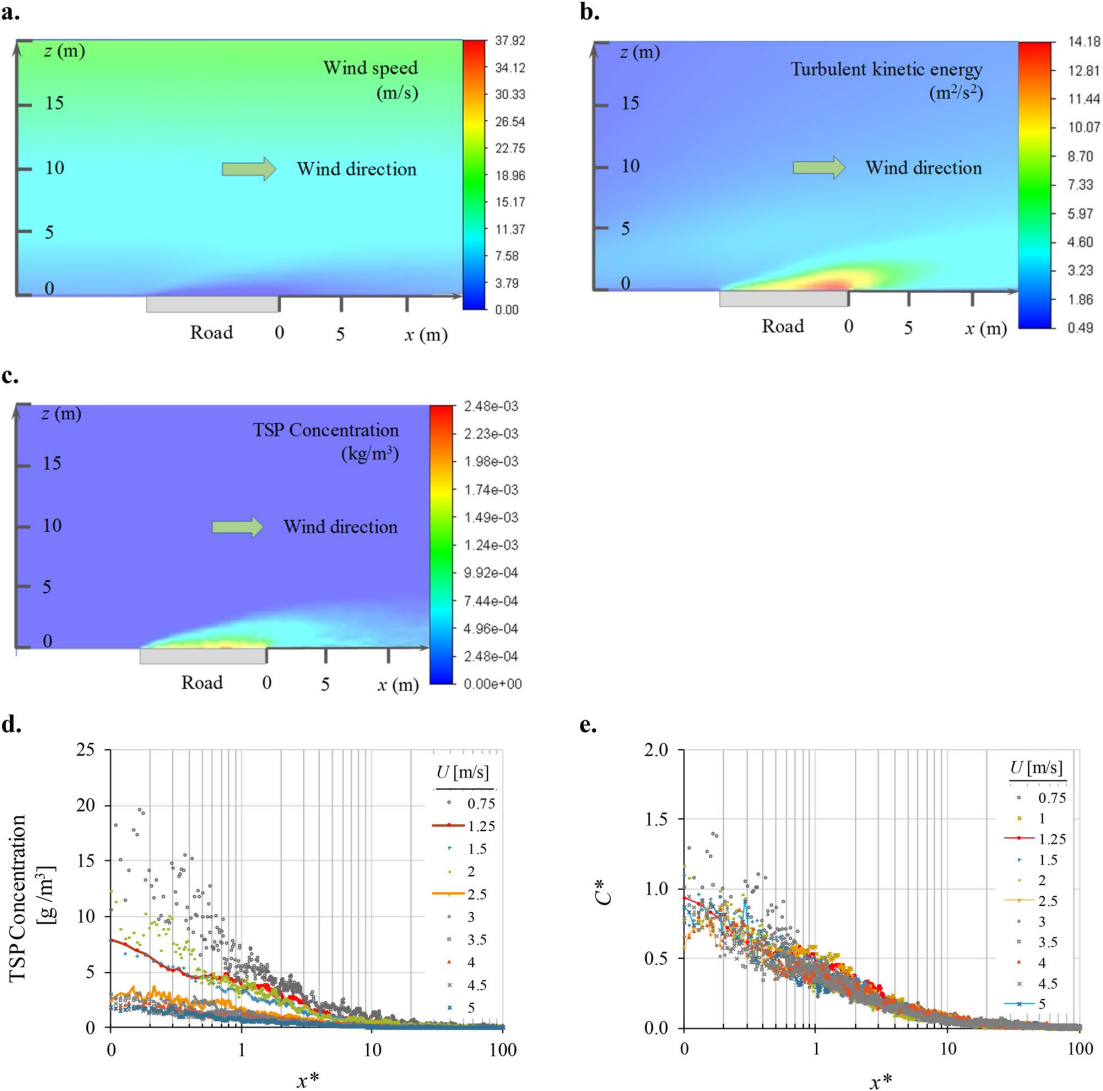
Results obtained by the NR-CFD model simulating the dispersion of TSP (1 < *d* < 34 *µ*m) emitted from a road located on a flat terrain without obstacles to the wind flow, under neutral atmospheric conditions. Resulting fields of (**a**) wind speed, (**b**) turbulent kinetic energy, and (**c**) TSP concentration. Resulting downwind ground TSP concentration as a function of wind speed when it is expressed as (**d**) *C* versus x* and (**e**) C* versus x*.

Then, we added the emissions of solid and gas-phase pollutants emitted from the road and observed their dispersion under different cases of atmospheric stability conditions. For the case of neutral conditions, Fig. 3a,b show the variations on the wind speed and turbulent kinetic energy, respectively, resulting from the mixing of the pollutants emitted from the road and the incoming wind flow. They show that the pollutants emitted at the road disturb the nearby wind flow and that those perturbations tend to disappear as the pollutants move away from the road.

Similarly, Fig. 3c illustrates the downwind TSP concentration resulting from the action of the wind dispersing the pollutants emitted from the road. This figure shows that the pollutant concentrations at the edge of the road reach their maximum values and then reduce toward a stable value far away from the road.

Figure 3d shows the downwind ground TSP concentrations as a function of wind speed, keeping the emission rate and the condition of atmospheric stability constant. It shows that the pollutant concentration (in this case, TSP) at the road edge reduces from ~ 15 to ~ 2.5 g m^−3^ when the wind speed increases from 0.75 to 5 m s^−1^. These results demonstrate the strong influence of wind speed on the resulting downwind pollutant concentration. Figure 3e shows the same results but now expressed in terms of C* and x*. It shows that all the profiles of downwind ground concentration shown previously in Fig. 3d now fall into a single universal profile. Previous results indicate that that C*, at any distance downwind the road, is independent of wind speed. It means that according to Eq. (13), the pollutant concentration (*C*) is inversely proportional to wind speed, which agrees with experimental observation. This result was first reported by Huertas and Prato^17^. Furthermore, they showed that C* is independent of the emission rate and the nature of the pollutant under consideration.

### Comparison with experimental results

**Figure 4:**
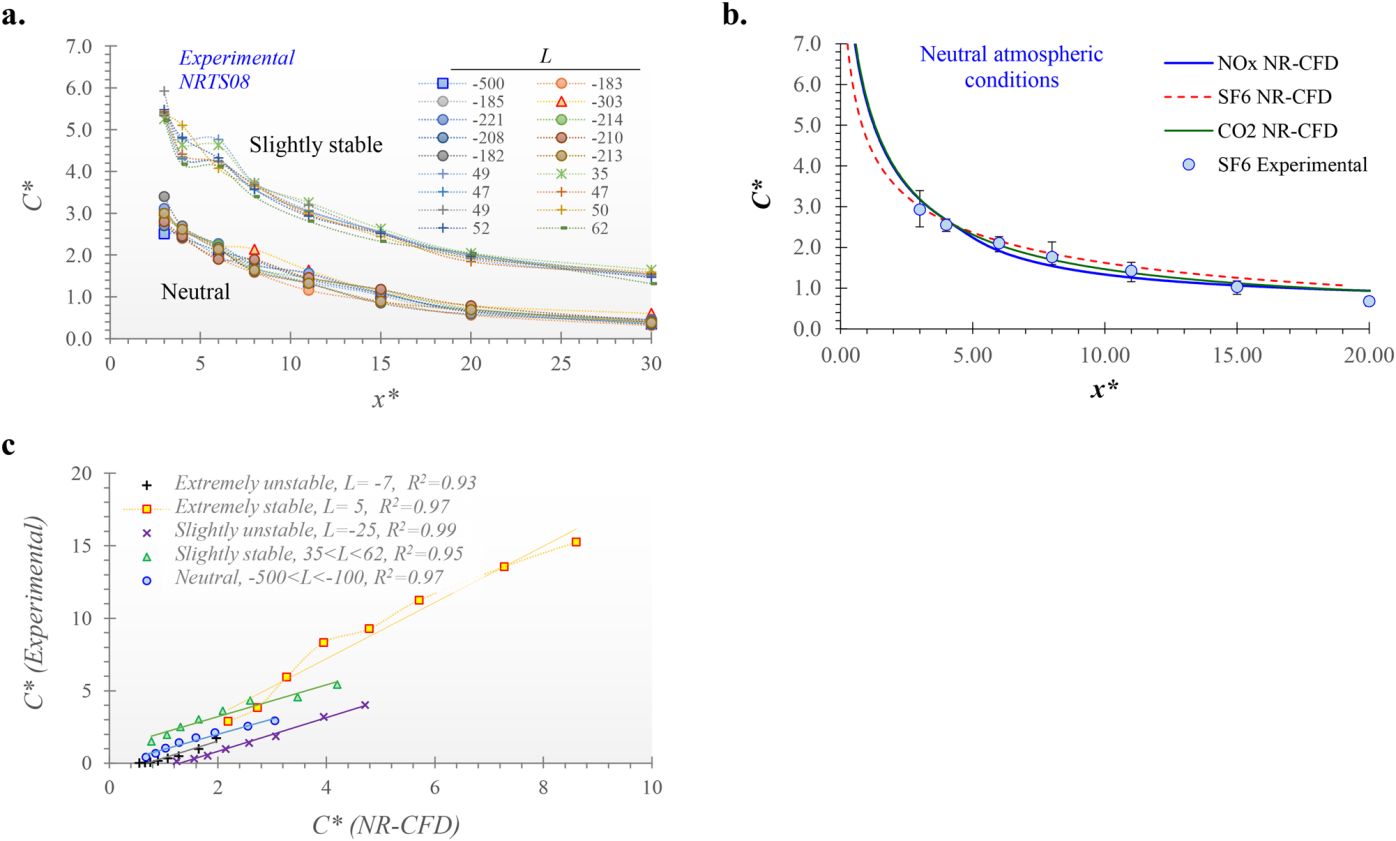
Comparison of NR-CFD results with experimental measurements obtained during the NRTS08 campaign under different atmospheric stability conditions^29^. (**a**) Experimental results obtained with SF6 under neutral and slightly stable conditions. (**b**) Comparison of SF_6_ experimental results with the obtained by the NR-CFD model simulating the dispersion of SF_6_, NOx and CO_2_ for the case of neutral atmospheric conditions. Vertical bars indicate the range of variation of experimental measurements. (**c**) Correlation analysis of experimental and NR-CFD results.

Figure 4.a shows the results obtained in the NRTS08 campaign when there was no barrier to the wind free flow for the cases when the atmospheric conditions were neutral (− 500 < *L* < − 100) and slightly stable (20 < *L* < 100). We used *Sc* = 0.21 for SF_6_. This figure confirms that, for neutral atmospheric conditions, when the downwind pollutant concentration is expressed in terms of dimensionless numbers, the observed profiles of C* versus x* are the same regardless of the wind speed or mass emission rate.

We then used the NR-CFD model to simulate the dispersion of SF_6_ under the same conditions that the experimental tests were carried out, using as input only the data points of speed and temperature measured by the meteorological station at 10 m high. Then, we compared these experimental results of C* with the results of C* obtained by the NR-CFD model for identical positions downwind the road and under similar atmospheric stability conditions (similar *L´s*). Figure 4.b compares the SF_6_ experimental results with the obtained by the NR-CFD model simulating the dispersion of SF_6_, NOx and CO_2_ for the case of neutral atmospheric conditions. Vertical bars indicate the range of variation of experimental measurements. We used *Sc* = 0.946 for NOx and *Sc* = 0.86 for CO_2_. In agreement with a previous work^17^, these results indicate that the C* versus x* is independent of the gas nature.

We repeated the comparison for results obtained under different atmospheric stability conditions and observed that the C* simulated and C* experimental are highly correlated (*R*^*2*^ > 0.95) and with slopes close to one (Fig. 4c). These results demonstrate the capacity of the NR-CFD model of reproducing short-term experimental measurements of pollutant dispersion under different atmospheric stability conditions.

### Effects of the atmospheric stability conditions on pollutant dispersion

Finally, we used the experimental results and the NR-CFD model to study the effects of the atmospheric conditions on the dispersion near roads of solid and gas phase pollutants.

Previously it was shown that when the downwind concentrations are expressed as C* vs x*, the resulting profile is independent of the wind speed, mass emission rate and the nature of the pollutant. That is, the use of the dimensionless numbers for concentration C* isolates the effect of wind speed, on pollutant dispersion. Then, from now on we will express results only in terms of these dimensionless variables.

**Figure 5:**
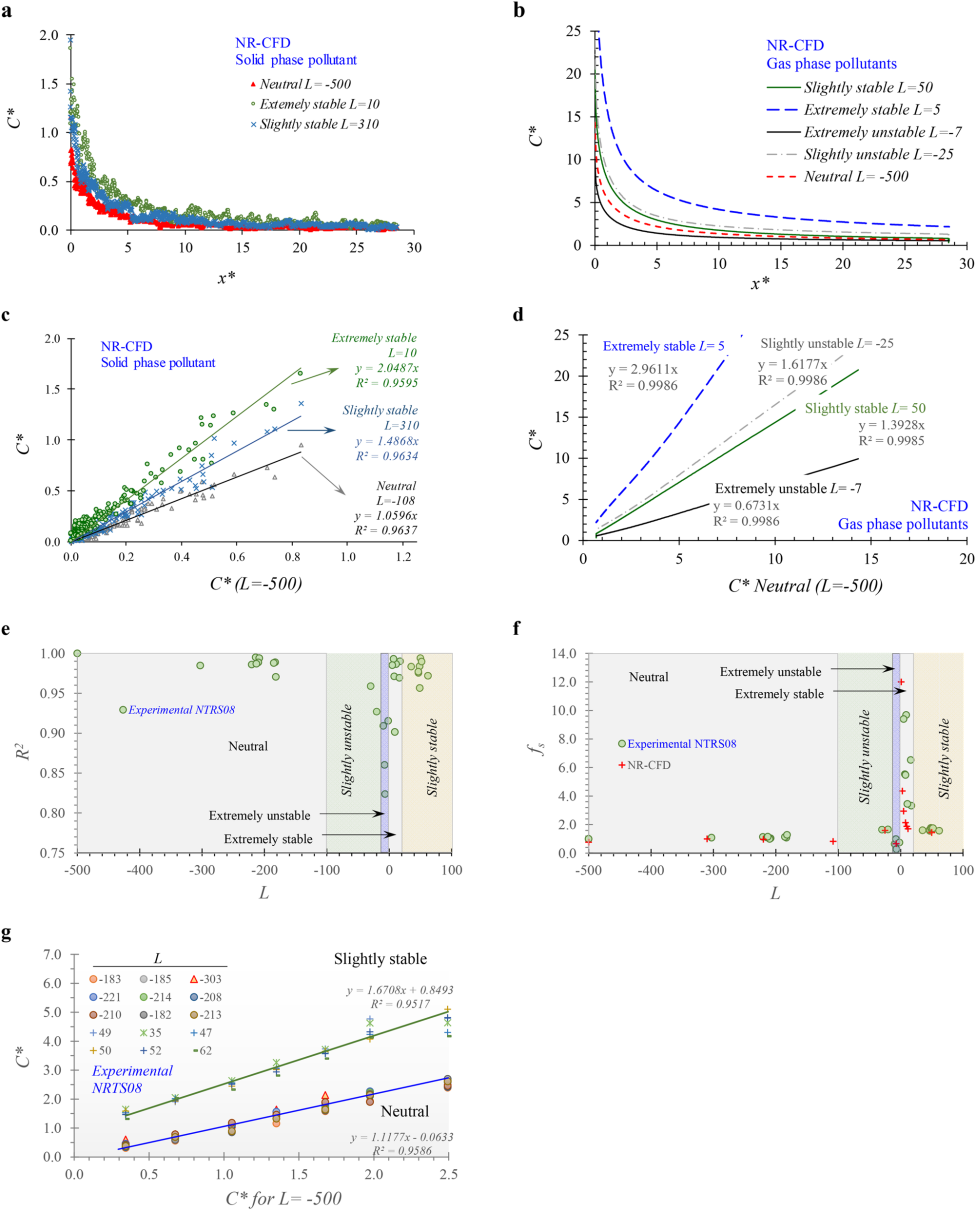
Effects of the atmospheric stability conditions on the dispersion of solid and gas-phase pollutants near roads. C* versus x* profiles for (**a**) solid-phase and (**b**) gas-phase pollutants under different atmospheric conditions; Correlation of the C* obtained under different stability conditions with the C* obtained under neutral conditions for (**c**) TSP and (**d**) gas-phase pollutants; (**e**) Coefficient of determination (*R*^2^) and (**f**) atmospheric stability dispersion factors (*f*_*s*_) obtained from the correlation analysis shown in (**d,g**) Correlation analysis among experimental measurements of SF_6_ concentrations under neutral and slightly stable conditions.

Figures 5a, b show the downwind ground concentration profiles (C* vs. x*) for solid (TSP) and gas-phase pollutants, respectively, obtained under different atmospheric stability conditions. Figures 5a,b show differences in the horizontal concentration profiles due to variations of the atmospheric stability conditions. Again, we highlight that these differences are due to the heat transfer processes that occur in the atmosphere under different stability conditions rather than the predominant effect of wind speed since the effects of wind speed are isolated from the analysis when the pollutant concentration is expressed as the dimensionless number for concentration (C*).

Aiming to quantify these differences, we selected the results obtained when the atmosphere was under neutral conditions and specifically when *L* = − 500 as a base case of comparison. Figures 5c shows that the downwind TSP concentration near roads obtained under different conditions of atmospheric stability is highly correlated with the one obtained under the neutral condition of atmospheric stability (*R*^*2*^ > 0.95). This figure shows that TSP dispersion under extremely stable atmospheric conditions (*L* = 10) leads to concentrations up to 2 times higher when compared to neutral conditions (slope ~ 2.05). Meanwhile, when the atmosphere is in a slightly stable condition (*L* = 310), TSP concentrations are ~ 48% greater than when the atmosphere is in a neutral condition. Finally, we observed that under any neutral condition (*L* < − 100), these slopes remained approximately equal to 1. We named these slopes, obtained from each correlation analysis, as the atmospheric stability dispersion factors (*f*_*s*_).

Similar results were obtained for the case of gas-phase pollutants. Figure 5d shows the results of the correlation analysis carried out among the gas-phase downwind concentrations obtained under different stability conditions compared to the ones obtained under a neutral condition of reference (*L* = − 500). For all cases, we observed a high correlation between the C* and the C* considered as reference (*R*^*2*^ > 0.99). Figure 5f shows the slopes (*fs*) obtained from the correlation analysis as functions of *L*. It shows that *fs* remains constant (*fs* ~ 1) for all simulations performed under neutral conditions (*L* < − 100). It increases to *fs* = 1.6 for the simulations carried out under slightly unstable conditions (− 100 < *L* < − 10) but decreases to *fs* ~ 0.66 for the simulations conducted under extremely unstable conditions (− 10 < *L* < 0). When the atmosphere is under extremely stable conditions (0 < *L* < 20) *fs* varies with *L* and reached values of up to *fs* = 12. Finally, when the atmosphere is under slightly stable conditions (*L* > 20) all the simulations showed an approximately constant value of *fs* = 1.6.

Aiming to double-check that these results were consistent with experimental observations, we revisited the subset of experimental data obtained in the NTRS08 campaign for the tests that were carried out without any barrier. We selected the test carried out when the atmosphere was under the most neutral conditions (*L* = − 500) and used the observed profile C* versus x* of this test as the base case of comparison. Then, for each test, we performed a correlation analysis between the C* measured during each test and the C* selected as the base case (Fig. 5g). As in the NR-CFD results, results showed a high correlation (*R*^*2*^ > 0.82) between the C* and the C* considered as the most neutral for all cases (Fig. 5e). Then, the *fs* obtained from the correlation analysis were plotted as function of *L* (Fig. 5f). This figure shows, qualitatively, that the experimentally obtained *fs* exhibit the same behavior obtained using the NR-CFD model. Finally, aiming to quantify the level of agreement between the values of *fs* obtained experimentally and numerically*,* we carried out a final correlation analysis among them. We found (no shown) a high agreement between them (slope = 1.11) with high consistency (*R*^*2*^ > 0.95).

### Use of the results obtained in this work for practical applications

As described before, for practical applications in environmental science, the results reported by the NR-CFD model are of great interest since they allow the prediction of short (~ 1 h) and long term (1 day–1 year) average concentration of a given pollutant at a given distance (*x*) from the road, under the varying conditions of wind speed (*U*), ambient temperature (*T*), and atmospheric stability conditions measured with a standard meteorological station. Nonetheless, the major drawback in the use of the NR-CFD model is the need of well-trained personnel to set up the NR-CFD model and the need of expensive software and hardware to run it for each time step.

We propose, as an approximation, the use of the universal solution reported in Fig. 3e (C* vs x*) and of the atmospheric stability dispersion factors, *fs* (Fig. 5f), as follow.

- For a given distance to the road (*x*), calculate the normalized distance to the road (x*) using Eq. (14).
- For each time step of data reported by the meteorological station:
  - Read from Fig. 3e the C* for the x* calculated in the previous step.
  - Read from Fig. 5f the value for *fs* according to the atmospheric stability condition.
  - Obtain the desired concentration *C*_*(x)*_ using Eq. (15)
    15 $$C_{\left( x \right)} = \frac{E}{{C_{{\left( {x*} \right)}}^{*} f_{s} U S_{c} }}$$
- Average the values obtained at each time step for *C*_*(x)*_.

When the wind blows in a direction different than the direction of the line perpendicular to the road, we suggest to use the strategy reported in Huertas et al.^17^, which is based on the consideration of the effective distance to the road *x*_*e*_ = *x*/*cos θ*, where *θ* is the angle between wind direction and the line perpendicular to the road.

## Conclusions

This work aimed to evaluate the influence of the atmospheric stability on the dispersion of pollutants emitted from an area source over a flat surface without any obstacle to the free wind flow.

Initially, a near road CFD based model to simulate the dispersion of pollutants in a reduced computational domain was implemented (NR-CFD model). It: (i) solves via CFD the governing equation that describe the dispersion of pollutants, (ii) uses the Monin–Obukhov Similarity (MOS) theory to specify the vertical profiles for wind speed and temperature as the inlet and ground boundary conditions, and (iii) uses the *k–ε* standard turbulence model calibrated experimentally for each atmospheric stability condition by Pieterse and Harms. This approach provides a rough approximation to the atmospheric stability conditions. However, it is not appropriate for the study of the atmosphere dynamics but can be used to obtain an approximation to the effects of the atmospheric dynamics on the dispersion of pollutants.

Then, aiming to isolate the effects of wind speed on pollutant dispersion, results were reported in terms of the dimensionless numbers of concentration (C*, Eq. 13) and distance to the road (x*, Eq. 14).

Results from the NR-CFD model agree with the experimental results obtained by the NOAA in 2008 observing the SF_6_ concentrations at several distances downwind a source line of SF_6_ under different atmospheric stability conditions. Experimental and analytical results show that when pollutant concentrations downwind the road are expressed as C* versus x*, the resulting profile is independent of wind speed, emission rates and the nature of the gas-phase pollutant. Therefore, variations observed on that profile could be attributed to variations on the atmospheric stability conditions rather than the sole effect of wind speed.

Experimental and analytical C* obtained under different atmospheric stability conditions are highly correlated (*R*^*2*^ > 0.82) to the C* obtained under neutral conditions. We named the slope obtained from the correlation analysis as the atmospheric stability factor (*fs*). Results show that *fs* remains constant (*fs* ~ 1) for neutral conditions (− 500 < *L* ≤ − 100). It increases to *fs* = 1.6 for slightly unstable conditions (− 100 < *L* ≤ − 10) and slightly stable conditions (*L* > 20) but decreases to *fs* ~ 0.66 for extremely unstable conditions (− 10 < *L* < 0). When the atmosphere is under extremely stable conditions (0 < *L* ≤ 20), *fs* varies with *L* and reached values of up to *fs* = 12.

Finally, for obtaining long-term average concentrations downwind the emission source, we proposed to assume a pseudo-steady-state approximation where at each time step (~ 1 h) the atmosphere is assumed to be under steady-state condition. For each time step, we suggest to use the results obtained under neutral conditions and affect the C* by *f*_*s*_ (Fig. 5f) to include the effects of the atmospheric stability conditions on pollutants concentration, where C* continues being the dimensionless number for pollutant concentration near roads obtained under neutral atmospheric conditions.

Even though these results indicate that they are valid for any non-reactive solid or gas-phase pollutant, we highlight that they are limited to the case of emission sources located on flat terrain without any obstacle of the free wind flow.

## Supplementary Information

Supplementary Information.

## References

[1] Koblitz T (2013). CFD Modeling of Non-Neutral Atmospheric Boundary Layer Conditions.

[2] Ashrafi K, Hoshyaripour GA (2008). A model to determine atmospheric stability and its correlation with CO concentration. Int. J. Environ. Ecol. Eng..

[3] Yuval TT, Raz R, Levi Y, Levy I, Broday DM (2020). Emissions vs. turbulence and atmospheric stability: A study of their relative importance in determining air pollutant concentrations. Sci. Total Environ..

[4] Seinfeld JH, Pandis SN (2006). Atmospheric Chemistry and Physics: From Air Pollution to Climate Change.

[5] Pal Arya S (1988). Introduction to Micrometeorology.

[6] Stull RB (2005). An Introduction to Boundary Layer Meteorology.

[7] Obukhov AM (1971). Turbulence in an atmosphere with a non-uniform temperature. Bound. Layer Meteorol..

[8] Pasquill F (1961). The estimation of the dispersion of windborne material. Meteorol. Mag..

[9] Turner D (1994). Workbook of Atmospheric Dispersion Estimates.

[10] Zoras S, Triantafyllou AG, Deligiorgi D (2006). Atmospheric stability and PM10 concentrations at far distance from elevated point sources in complex terrain: Worst-case episode study. J. Environ. Manag..

[11] Finn D (2009). Tracer studies to characterize the effects of roadside noise barriers on near-road pollutant dispersion under varying atmospheric stability conditions. Atmos. Environ..

[12] Wang F (2016). Quantifying stability influences on air pollution in Lanzhou, China, using a radon-based "stability monitor": Seasonality and extreme events. Atmos. Environ..

[13] Fritts DC, Wang L, Geller MA, Lawrence DA, Werne J, Balsley BB (2016). Numerical modeling of multiscale dynamics at a high Reynolds number: Instabilities, turbulence, and an assessment of ozmidov and thorpe scales. J. Atmos. Sci..

[14] Gopalan H, Gundling C, Brown K, Roget B, Sitaraman J, Mirocha JD, Miller WO (2014). A coupled mesoscale-microscale framework for wind resource estimation and farm aerodynamics. J. Wind Eng. Ind. Aerodyn..

[15] Haupt SE, Kosovic B, Shaw W, Berg LK, Churchfield M, Cline J, Sever G (2019). On bridging a modeling scale gap: Mesoscale to microscale coupling for wind energy. Bull. Am. Meteorol. Soc..

[16] Alinot C, Masson C (2005). k-ɛ model for the atmospheric boundary layer under various thermal stratifications. J. Sol.Energy Eng..

[17] Huertas JI, Prato Sánchez DF (2019). An experimental and numerical study of air pollution near unpaved roads. Air Qual. Atmos. Health.

[18] Steffens JT, Heist DK, Perry SG, Zhang KM (2013). Modeling the effects of a solid barrier on pollutant dispersion under various atmospheric stability conditions. Atmos. Environ..

[19] Wang YJ, Zhang KM (2009). Modeling near-road air quality using a computational fluid dynamics model, CFD-VIT-RIT. Environ. Sci. Technol..

[20] Barratt R (2001). Atmospheric Dispersion Modelling: An Introduction to Practical Applications.

[21] Pieterse JE, Harms TM (2013). CFD investigation of the atmospheric boundary layer under different thermal stability conditions. J. Wind Eng. Ind. Aerodyn..

[22] Franke, J., Hellsten, A., Schlunzen, H. & Carissimo, B. E. *Best Practice Guideline for the CFD Simulation of Flows in the Urban Environment. Cost Action 732: Quality Assurance and Improvement of Microscale Meteorological Models* (COST, 2007).

[23] Stull R (2016). Practical Meteorology: An Algebra-Based Survey of Atmospheric Science.

[24] Blocken B, Gualtieri C (2012). Ten iterative steps for model development and evaluation applied to Computational Fluid Dynamics for environmental fluid mechanics. Environ. Model. Softw..

[25] De Visscher A (2013). Air Dispersion Modeling. Foundations and Applications.

[26] Blocken B, Stathopoulos T, Carmeliet J (2007). CFD simulation of the atmospheric boundary layer: Wall function problems. Atmos. Environ..

[27] ANSYS. *ANSYS FLUENT 12.0 Theory Guide*. https://www.afs.enea.it/project/neptunius/docs/fluent/html/th/main_pre.htm (2020).

[28] Huertas JI, Huertas ME, Solis C (2012). Characterization of airborne particles in an open pit mining region. Sci. Total Environ..

[29] ANSYS. *ANSYS FLUENT 12.0 User's Guide. *https://www.afs.enea.it/project/neptunius/docs/fluent/html/ug/main_pre.htm (2012).

[30] Clawson KL (2010). Tracer studies to characterize the effects of roadside noise barriers on near-road pollutant dispersion under varying atmospheric stability conditions. Atmos. Environ..

