# Supplementary material for: Numerical approximation to the effects of the atmospheric stability conditions on the dispersion of pollutants over flat areas
Source: Sci Rep. 2021 Jun 2;11:11566. doi: 10.1038/s41598-021-89200-9 (PMC8172944; doi:10.1038/s41598-021-89200-9)
Supplement: Supplementary file 1 — Supplementary Information. [file 41598_2021_89200_MOESM1_ESM.docx]

**Numerical approximation to the effects of the atmospheric stability conditions on the dispersion of pollutants over flat areas**

J.I. Huertas^1,*^; D.S. Martinez^1^; D.F. Prato^2^

*^1^ Energy and climate change research group, School of Engineering and Science, Tecnológico de Monterrey, Mexico.*

*^2^ Latin-American Center for Innovation and Logistics (CLI), Bogotá, Colombia*

** Corresponding author.* [*jhuertas@tec.mx*](mailto:jhuertas@tec.mx)

**Symbols and acronyms**

|  | ***Description*** | ***Units*** |
| --- | --- | --- |
| *C* | Pollutant concentration | *μg m^-3^* |
| *C** | Normalized pollutant concentration | *-* |
| *k* | Von Karman universal constant | *-* |
| *R^2^* | Coefficient of determination | *-* |
| *u* | Local wind speed at height *z* | *m s^-1^* |
| *u´* | Instantaneous fluctuating random speed of the continuous phase | *m s^-1^* |
| *u_p_* | Local particle speed in the *x* direction | *m s^-1^* |
| *u** | Friction speed | *m s^-1^* |
| *U* | Mean wind speed in the *x* direction measured at 10 m above ground | *m s^-1^* |
| *W* | Road width | *m* |
| *x* | Distance to the edge of the road (downwind) | *m* |
| *x** | Normalized distance to the road edge | *-* |
| *z* | Height | *m* |
| *z_o_* | Surface roughness | *m* |
| *ε* | Eddy viscosity | *m^2^ s^-3^* |
| $\rho$*,* $\rho_{p}$ | Fluid and particle density | *kg m^-3^* |
| $\mu$ | Fluid molecular viscosity | *kg m^-1^s^-1^* |
| SBL | Near surface atmospheric boundary layer | *-* |
| CFD | Computational fluid dynamics | *-* |
| NR-CFD | Near road CFD model | *-* |
| TSP | Total suspended particles (particles with aerodynamic diameter *d* < ~30μm) | *-* |
